# Quantitative immunofluorescence microscopy of subcellular GLUT4 distribution in human skeletal muscle: effects of endurance and sprint interval training

**DOI:** 10.14814/phy2.12085

**Published:** 2014-07-22

**Authors:** Helen Bradley, Christopher S. Shaw, Philip L. Worthington, Sam O. Shepherd, Matthew Cocks, Anton J. M. Wagenmakers

**Affiliations:** 1 School of Sport, Exercise and Rehabilitation Sciences, University of Birmingham, Birmingham, UK; 2 Institute of Sport, Exercise and Active Living (ISEAL), Victoria University, Melbourne, Vic., Australia; 3 Computational Biology, Discovery Sciences, AstraZeneca, Alderley Park, Macclesfield, UK; 4 Research Institute for Sport and Exercise Sciences, Liverpool John Moores University, Liverpool, UK

**Keywords:** Glucose uptake, insulin sensitivity, skeletal muscle

## Abstract

Increases in insulin‐mediated glucose uptake following endurance training (ET) and sprint interval training (SIT) have in part been attributed to concomitant increases in glucose transporter 4 (GLUT4) protein content in skeletal muscle. This study used an immunofluorescence microscopy method to investigate changes in subcellular GLUT4 distribution and content following ET and SIT. Percutaneous muscle biopsy samples were taken from the *m. vastus lateralis* of 16 sedentary males in the overnight fasted state before and after 6 weeks of ET and SIT. An antibody was fully validated and used to show large (> 1 *μ*m) and smaller (<1 *μ*m) GLUT4‐containing clusters. The large clusters likely represent trans‐Golgi network stores and the smaller clusters endosomal stores and GLUT4 storage vesicles (GSVs). Density of GLUT4 clusters was higher at the fibre periphery especially in perinuclear regions. A less dense punctate distribution was seen in the rest of the muscle fibre. Total GLUT4 fluorescence intensity increased in type I and type II fibres following both ET and SIT. Large GLUT4 clusters increased in number and size in both type I and type II fibres, while the smaller clusters increased in size. The greatest increases in GLUT4 fluorescence intensity occurred within the 1 *μ*m layer immediately adjacent to the PM. The increase in peripheral localisation and protein content of GLUT4 following ET and SIT is likely to contribute to the improvements in glucose homeostasis observed after both training modes.

## Abstract (precis)

e12085

This paper first describes the development of a novel confocal immunofluorescence microscopy method that allows quantitation of GLUT4 content in the plasma membrane and 1 µm layers below it in a muscle fibre‐type specific manner. Skeletal muscle biopsies obtained from sedentary young men before and after 6 weeks of traditional endurance training (ET) and sprint interval training (SIT) were then analysed to show for the first time increases in large and small GLUT4 clusters with greater increases in the layer within 1 µm of the plasma membrane, which is the layer from which most GLUT4 fusion events emanate. This training‐induced redistribution is likely to contribute to the increase in insulin sensitivity seen following both ET and SIT.

## Introduction

Skeletal muscle is the primary site of insulin‐mediated glucose uptake (Katz et al. 1983; Wasserman et al. 2011) and is therefore important in determining whole body glucose disposal rates. Glucose transporter 4 (GLUT4) is the major glucose transporter isoform expressed in human skeletal muscle and is the primary insulin‐responsive glucose transporter (Mueckler 1994). GLUT4 is distributed between intracellular storage clusters associated with intracellular membranes, the plasma membrane (PM) and T‐tubule membranes. Following insulin stimulation, GLUT4 translocation and fusion with both the PM and T‐tubule membranes leads to increases in surface membrane GLUT4 content which is assumed to be the major mechanism by which physiological increases in plasma insulin stimulate glucose uptake into muscle cells (Bell et al. 1993; Ploug et al. 1998; Lauritzen et al. 2006, 2008; Lizunov et al. 2012).

Experimental increases in muscle GLUT4 content are known to coincide with a higher capacity for insulin stimulated glucose uptake, as demonstrated in animal models overexpressing GLUT4 in skeletal muscle (Hansen et al. 1995; Ren et al. 1995; Leturque et al. 1996; Tsao et al. 1996). Similarly, the increases in insulin stimulated glucose uptake rates that are seen following endurance exercise training (ET) and various forms of high‐intensity interval training (HIT) occur in parallel with robust increases in total skeletal muscle GLUT4 protein content (Houmard et al. 1991; Hughes et al. 1993; Dela et al. 1996; Phillips et al. 1996; Cox et al. 1999; Daugaard et al. 2000; Kristiansen et al. 2000; Burgomaster et al. 2007; Babraj et al. 2009; Little et al. 2010; Richards et al. 2010; Hood et al. 2011). In addition, a single exercise bout has been shown to increase total crude membrane GLUT4 protein content (Kraniou et al. 2006).

Studies in adipose cells have revealed that GLUT4 is stored in 50–70 nm insulin responsive vesicles termed GLUT4 storage vesicles (GSVs) and endosomal membrane clusters estimated to be approximately 150–200 nm (Larance et al. 2008; Bogan and Kandror 2010; Stockli et al. 2011; Bogan 2012). GSVs in adipocytes are formed by insulin stimulated budding of GLUT4 clusters that are associated with endosomal and trans‐Golgi network (TGN) membranes (Larance et al. 2008; Bogan and Kandror 2010; Bogan 2012). The budding is then followed by GSV translocation, docking and fusion with the PM to thus increase the glucose uptake capacity (Foley et al. 2011). Studies in whole single fibres of rat soleus muscle using both confocal immunofluorescence microscopy and electron microscopy (EM) combined with immunogold labelling have also demonstrated the presence of GLUT4 in TGN membranes, endosomal membranes and GSVs (Rodnick et al. 1992; Ploug et al. 1998; Lauritzen et al. 2008; Lizunov et al. 2012). Immunofluorescence microscopy studies applied to muscle fibres of rodents have defined TGN GLUT4 stores as >1 *μ*m in diameter (Lauritzen et al. 2008), while based on EM images endosomes appear to be smaller than TGN stores (Ploug et al. 1998) but larger than GSVs which have been reported to be as small as 40 nm (Lizunov et al. 2012).

In the basal state, GLUT4 is predominantly located at the muscle fibre periphery in rodent muscle, especially in perinuclear regions (Ploug et al. 1998; Lauritzen et al. 2006). A less dense punctate distribution was seen in the rest of the muscle fibre (Ploug et al. 1998; Lauritzen et al. 2006). The T‐tubule membranes are invaginations of the PM which provide a high‐ surface area for insulin delivery to the muscle cell and glucose uptake and are observed as cross‐striations in immunofluorescence imaging of longitudinally oriented muscle fibres (Ploug et al. 1998). Cross‐striations of GLUT4 staining associated with T‐tubule membranes were weak and irregular in rodent muscle in the basal state (Ploug et al. 1998; Lauritzen et al. 2006, 2008).

Following insulin stimulation of rodent muscle, the large GLUT4 clusters did not translocate but remained stationary and were locally depleted (Lauritzen et al. 2008). Furthermore, total internal reflection fluorescence (TIRF) microscopy studies in isolated mouse muscle showed that more than 80% of insulin‐stimulated fusion events emanated from stationary vesicles that had been pretethered at the PM or T‐tubule membranes prior to stimulation, with only 10% of all GLUT4 structures shown to be mobile (Lizunov et al. 2012). Taken together, these data suggest that GLUT4 movement following insulin stimulation occurs over a short distance and implies that basal state GLUT4 localisation close to the PM could impart a benefit for insulin action on glucose uptake.

In earlier studies, GLUT4 translocation was primarily investigated using subcellular fractionation techniques in both rodent (Klip et al. 1987; Douen et al. 1990; Marette et al. 1992) and human skeletal muscle (Goodyear et al. 1996; Kennedy et al. 1999) through quantitation of GLUT4 content in PM fractions. Contamination of the isolated PM fractions can occur with myofibrillar proteins and with other intracellular membrane fractions with a high‐GLUT4 content (Fazakerley et al. 2009). A further limitation is the inability to discriminate between GLUT4 just adjacent to the PM and GLUT4 fully incorporated into the PM after docking and fusion of GSV's and, therefore, able to transport glucose (Schertzer et al. 2009). In vivo model systems have been developed in which mice express GLUT4‐HA for confirmation of GLUT4 insertion into the PM (Fazakerley et al. 2009; Schertzer et al. 2009). However, in human skeletal muscle in vivo transient or chronic expression of tagged‐GLUT4 for experimental purposes is not technologically possible. New methods, beyond PM fractionation must be developed to enable further investigation of GLUT4 localisation and trafficking in human skeletal muscle.

This study aimed to develop a confocal immunofluorescence microscopy method to investigate whether GLUT4 localisation in human skeletal muscle is similar to the distribution previously observed in rodent muscle with large and smaller GLUT4 stores occurring in a higher density in the perinuclear area and close to the PM. To achieve this, a GLUT4‐specific antibody was first robustly validated for use in immunofluorescence microscopy on human muscle. The newly developed method was subsequently applied to human skeletal muscle samples taken in an earlier study from our laboratory (Cocks et al. 2013; Shepherd et al. 2013). We investigated the hypothesis that ET and SIT increase the number and size of the large and small GLUT4 clusters in peripheral regions of the muscle fibre which dictate the amount of GLUT4 available for translocation and impart a benefit upon insulin‐mediated glucose uptake.

## Methods

### Study location

The human studies were executed in the School of Sport and Exercise Sciences at the University of Birmingham, UK. The plasma and muscle samples were analysed in part at the University of Birmingham, UK, Liverpool John Moores University, UK, and the Computational Biology Group of AstraZeneca, Alderley Park, UK.

### Ethics, recruitment and informed consent

Sixteen sedentary, but otherwise healthy, males (mean ±SEM, age 21 ± 1 years, BMI 23.7 ± 0.8 kg·m^−2^, VO_2 max_ 41.8 ± 2.2 mL·min^−1^·kg^−1^) all gave written informed consent before being randomly divided into two age‐, BMI‐ and VO_2max_‐matched groups to complete 6 weeks of either ET or SIT. The immunofluorescence protocol was developed and the GLUT4 antibody validated in skeletal muscle from 5 lean healthy males. Ethical approval was granted for the study by the Black Country Research Ethics Committee and all procedures conformed to the Helsinki declaration.

### Training and testing

All procedures for pre‐ and post‐training experiments and training interventions have been previously reported (Cocks et al. 2013; Shepherd et al. 2013). Briefly, all participants underwent a fasting muscle biopsy and a 2 h oral glucose tolerance test prior to and following 6 weeks of training. All post‐training experiments were performed at least 48 h following the last training bout. On a separate day, participants performed an incremental exercise test to exhaustion on an electronically braked cycle ergometer. ET consisted of five sessions per week of cycling on an electronically braked cycle ergometer at 65% of VO_2max_, with session duration increasing from 40 min to 60 min over the 6 week training period. SIT subjects completed three sessions per week each consisting of 4–6 Wingate tests (increasing over the 6 week training period). Each Wingate required a 30 sec “all out” sprint against a resistance of 0.075 kg/kg body mass followed by 4.5 min low‐intensity cycling on an electronically braked cycle ergometer.

### Sample collection and preparation

All skeletal muscle samples were taken from the *m. vastus lateralis* using the percutaneous needle biopsy technique (Bergstrom 1975). Immediately after collection, muscle biopsy samples were blotted to remove excess blood and any visible collagen or fat was discarded. Part of the sample was embedded in Tissue Tek OCT compound (Sakura) and immediately frozen in liquid nitrogen‐cooled isopentane (Sigma Aldrich, St. Louis, MO). The sample was then transferred to an aluminium cryotube (Caltag Medsystems, Buckingham, UK) for storage at ‐80°C. The remaining sample was snap frozen in liquid nitrogen to be used for Western blot analysis and stored at ‐80°C. For immunofluorescence staining frozen muscle biopsy samples were cryosectioned using a microtome within a cryostat (Bright Instrument Company Limited, Huntingdon, UK) to a thickness of 5 *μ*m onto uncoated glass microscope slides (VWR International, Leicester, UK). Pre‐ and post‐training sections to be compared with each other were cut onto the same slide and slides for each participant were prepared in triplicate. Slides were either stained within 1 h of sectioning or stored at ‐20°C. All slides for each participant were treated in an identical manner. Following storage slides were left at room temperature for 30 min prior to staining. For western blotting snap frozen muscle biopsy samples were homogenised in liquid nitrogen using a pestle and mortar. Homogenised sample was then mixed in 1x RIPA buffer (Cell Signaling Technology, Danvers, MA) with protease (Roche, Basel, Switzerland) and phosphatase inhibitors (ThermoScientific, Waltham, MA) on ice for 4 h on a shaker. Sample was then further homogenised using a polytron tissue homogeniser (IKA T25, Ultra‐Turrax, Stauffen, Germany) prior to centrifugation at 10,000 *g* for 20 min at 4°C. A small portion of supernatant was used to determine protein concentration (BCA protein assay kit, ThermoScientific) and the remaining supernatant was frozen at ‐80°C.

### Antibody validation

For Western blotting, protein extract (60 *μ*g) was loaded onto a 7.5% resolving gel (Biorad, Hertfordshire, UK) with 5 *μ*L molecular weight marker (Biorad) and run for 1 h at 40 mA in 1x Tris/Glycine/SDS buffer (Biorad). Proteins were transferred to a PVDF membrane (Biorad) for 2 h at 350 mA in 1x Tris/Glycine buffer (Biorad) with 20% methanol. The membrane was blocked for 1 h in 5% nonfat dry milk (Cell Signaling Technology) made up in tris‐buffered saline (50 mmol/L Tris‐HCl (Sigma Aldrich), 150 mmol/L NaCl (Sigma Aldrich) with 0.05% tween 20 (Sigma Aldrich) (TBST) and then incubated overnight at 4°C in 1:2000 dilution of GLUT4 primary antibody (ab654, Abcam, Cambridge, UK) in 5% milk. Membrane was subsequently washed 3 times for 10 min in TBST and blocked again for 1 h in 5% milk prior to incubation in 1:2000 dilution of goat–anti‐rabbit IgG HRP‐conjugated secondary antibody (Cell Signaling Technology) in 5% milk for 1 h. Membrane was then washed twice in TBST and once in TBS without tween. Membrane was incubated in ECL substrate (GE Healthcare, Fairfield, CT) for 5 min prior to exposure to film (GE Healthcare) and development of film. To confirm GLUT4 antibody specificity and selectivity in an immunofluoresence application experiments were carried out onsite at AstraZeneca in an L6 cell line overexpressing GLUT4 with a myc protein tag (GLUT4‐myc) and nontransfected L6 cells. L6‐GLUT4myc cells were purchased by AstraZeneca from Dr. Amira Klip and Dr. Philip Bilan (Department of Biochemistry, University of Toronto) and have been described previously (Kanai et al. 1993; Wang et al. 1998). GLUT4‐myc and nontransfected cells (20,000 per well) were differentiated to myotubes and were fixed in 4% formaldehyde for 5 min on ice and then kept for 20 min at room temperature. Cells were washed twice in PBS and 100 *μ*L 0.1M glycine was added to quench the formaldehyde reaction. Cells were permeabilised in 100 *μ*L 0.1% triton X‐100 in PBS for 5 min at room temperature and then washed once in PBS. Cells were blocked for 1 h at room temperature in 3% BSA in PBS and then incubated in 25 *μ*L 1:50 GLUT4 antibody in 3% BSA with 1:50 mouse anti‐myc (Sigma Aldrich) for 1 h at room temperature. To confirm secondary antibody specificity, a negative control was also performed where the GLUT4 primary antibody was omitted. Cells were then washed once in PBS and secondary antibodies were applied for 40 min at room temperature in 3% BSA with a 1:500 Hoechst 33342 (Invitrogen, Paisley, UK) counterstain for cell nuclei. Goat anti‐rabbit IgG 594 (1:500) was used to detect GLUT4 primary antibody and 1:500 goat anti‐mouse IgG 488 was used to detect myc primary antibody. Cells were then washed three times in PBS and left in PBS for viewing. Viewing was completed using ImageXpress with a 10x objective and contrast was adjusted consistently across all images. To confirm specificity of the GLUT4 antibody for the immunogen peptide, the antibody was incubated with saturating concentrations (5 times higher than the antibody concentration) of the GLUT4 immunogen peptide (ab115831, Abcam) for 24 h at 4°C, prior to application onto human skeletal muscle sections in place of primary antibody. All other stages of the staining protocol remained identical and the competition staining was completed alongside a positive control with primary antibody and a negative control in which primary antibody was omitted completely.

### Immunofluorescence staining protocol

The immunofluorescence staining protocol was optimised for analysis of GLUT4 in human skeletal muscle and negative controls were performed to confirm there were no problems with antibody cross reactivity. Muscle sections were fixed for 5 min in 75% acetone 25% ethanol. Subsequently sections were washed 3 times for 5 min in phosphate‐buffered saline (PBS, 137 mmol/L sodium chloride, 3 mmol/L potassium chloride, 8 mmol/L sodium phosphate dibasic, 3 mmol/L potassium phosphate monobasic). GLUT4 antibody (rabbit IgG, Abcam) was applied to the sections at a dilution of 1:200 in 5% normal goat serum (NGS, Invitrogen) for 2 h at room temperature. The antibody used binds the cytosolic C terminal of GLUT4, as such the GLUT4 visualised in this study represents both surface membrane‐bound and intracellular GLUT4. GLUT4 primary antibody was combined with either dystrophin (mouse IgG_2b_, 1:400, Sigma Aldrich), dihydropyridine receptor (DHPR; mouse IgG_2a_, 1:25, Abcam) or myosin heavy chain (MHCI; mouse IgM, 1:100, developed by Dr. Blau, DSHB, USA) antibodies. Following primary antibody incubation sections were washed 3 times for 5 min in PBS. Secondary antibodies were applied to sections for 30 min at room temperature at a dilution of 1:200 in PBS. GLUT4 antibody was targeted with goat anti‐rabbit IgG 488 (Invitrogen), dystrophin with goat anti‐mouse IgG_2b_ 594 (Invitrogen) or goat anti‐mouse IgG_2b_ 633 (Invitrogen), DHPR with goat anti‐mouse IgG_2a_ 594 (Invitrogen) and MHCI with goat anti‐mouse IgM 594 (Invitrogen). Secondary antibody combinations specific to each figure are described in the figure legends. DAPI (Sigma Aldrich) staining for cell nuclei was added to the secondary antibody at a 0.5 *μ*g·mL^−1^ concentration. After secondary antibody incubation sections were washed 3 times for 5 min in PBS and coverslips were mounted with 20 *μ*L mowiol; 6 g glycerol (Sigma Aldrich), 2.4 g mowiol 4‐88 (Sigma Aldrich) and 0.026 g 1,4‐diazobicyclo‐[2,2,2]‐octane (DABCO) (Sigma Aldrich) dissolved in 18 mL 0.2 mol/L Tris‐buffer (pH 8.5) (Sigma Aldrich).

### Image capture

Widefield image capture was completed using a Nikon E600 microscope coupled to a SPOT RT KE colour three shot CCD camera (Diagnostic Instruments Inc., Sterling Heights, MI). FITC, Texas Red and DAPI UV excitation filters were used to visualise the Alexa Fluor 488 and 594 fluorophores, and DAPI nuclear stain, respectively. Confocal image capture was completed using an inverted confocal microscope (Leica DMIRE2, Leica Microsystems, Wetzlar, Germany) with a 63x oil immersion objective (1.4 NA). In addition the pre‐ and post‐training images were captured at a 2x zoom. Individual fibres were captured per image and were chosen at random during image capture with a selection routine only considering the fibre type, not the GLUT4 labelling. AlexaFluor 488 fluorophores (emission range 475–655 nm) were excited with a 488 nm line of the argon laser for excitation and 498‐571 nm emission filter. Alexa Fluor 594 fluorophores (emission range 580‐751 nm) were excited with the 594 nm line of the helium‐neon laser for excitation and 601‐713 nm emission filter. Alexa Fluor 633 fluorophores (emission range 610‐801 nm) were excited with the 633 nm line of the helium‐neon laser for excitation and 639‐734 nm emission filter.

### Image quantitation

For each subject, three slide replicates, each with a pre‐training and a post‐training section, were stained, imaged and quantified. At least 5 type I fibre images and 5 type II fibre images were captured per section, therefore for each subject at least 30 images (15 type I fibres and 15 type II fibres) were analysed for both pre‐ and post‐training.

For quantitation of GLUT4 in the whole muscle fibre, all image processing and analysis was carried out in ImagePro Plus 5.1 and was kept consistent between images. GLUT4 fluorescence intensity was quantified by measuring the signal intensity within the intracellular regions of a mask created by the dystrophin stain in a fibre type specific manner as determined from the MHCI stain. No image processing was carried out prior to intensity analysis. Intracellular spot number and staining area per fibre was quantified by setting uniform threshold intensity and size values to identify spots within intracellular regions of the dystrophin mask in a fibre type specific manner. In rodent muscle large GLUT4 stores which were stationary were greater than 1 *μ*m in diameter. The small, mobile stores in rodent muscle were reported as <1 *μ*m in diameter (Lauritzen et al. 2008). Large and small spots in this study were determined by limiting the threshold size values, whereby spots >1 *μ*m in diameter were classed as large spots and spots <1 *μ*m in diameter were classified as small spots. The mean size of spots was calculated by dividing the total area of spot staining by the number of spots per fibre. An ImagePro Plus no neighbour deconvolution algorithm and a HiGauss filter were applied to GLUT4 images prior to spot detection.

**Figure 1.**
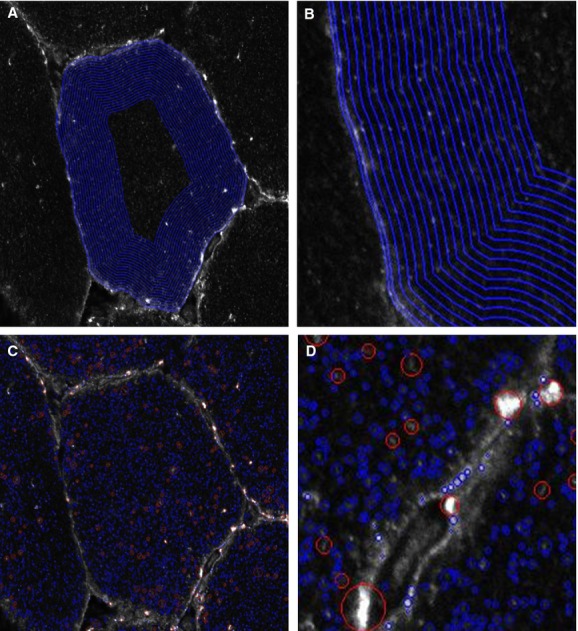
Representation of image analysis method to assess GLUT4 in the PM layer and 1 *μ*m intracellular layers. (A) Final dystrophin segmentation overlaid on GLUT4 image with PM layer and intracellular layers marked. (B) Detail showing 5 pixel PM layer and 20 1 *μ*m thick intracellular layers. (C) Spot detection and post‐processing applied to identify large (red) and small (blue) GLUT4 spots. (D) Detail showing identification of large (red) and small (blue) GLUT4 spots. This novel analysis method provides a vital tool to investigate changes in the main GLUT4 stores in response to acute exercise and training, focussing on the PM layer and first intracellular layers as these are the sites from which fusion events emanate.

For quantitation of GLUT4 in the PM layer (dystrophin‐stained region) and 1 *μ*m layers in from the PM all image processing and analysis was carried out in MATLAB (v. 2012b, The MathWorks Inc., Natick, MA, 2012) using a bespoke image analysis algorithm. The analysis method is depicted in Figure 1. In brief the steps of the analysis algorithm were to segment the fibres in the dystrophin image using the active contour, or snake, approach (Kass et al. 1988) to approximately find the mid‐point of the PM, and use a distance map from the contour to generate a 2.5 pixel thick region to cover the dystrophin‐stained region. This region has been designated the PM layer. Subsequently twenty 1 *μ*m thick layers were generated inside the fibre, again using the distance map. GLUT4 large and small spots were identified using intensity and size thresholds within each region (large >1 *μ*m, small <1 *μ*m diameter).

### Western blotting for GLUT4 content

Equal total protein quantities (60 *μ*g) of all ET and SIT samples were separated by electrophoresis on 12% polyacrylamide gels in 1x Tris/Glycine/SDS buffer (National Diagnostics) for 20 min at 80 V and 2 hr at 120 V. Proteins were then transferred to nitrocellulose membrane using Invitrogen iblot apparatus (Invitrogen) and Invitrogen iblot gel transfer stacks (Invitrogen). Following transfer membranes were washed briefly in phosphate buffered saline (PBS, tablets, Merck Millipore, Germany) with 0.1% tween‐20 (Sigma Aldrich) and bands were visualised with Ponceau S to confirm successful transfer. Ponceau S staining was removed with 3 washes for 2 min in PBST. Membranes were blocked for 1 h in 5% milk (dried skimmed milk, Marvel) and incubated overnight at 4°C on a shaker in 1: 2000 GLUT4 primary antibody in 5% milk. Following incubation membranes were washed 4 times for 15 min in PBST and incubated in 1:15,000 goat anti‐rabbit IgG HRP secondary antibody (DAKO, Glostrup, Denmark) in 5% milk at room temperature for 1 h. Membranes were again washed 4 times for 15 min in PBST and were incubated for 5 min in ECL substrate (GE Healthcare) prior to exposure of membrane to film (Kodak, UK). Film was developed using a Xograph Imaging Systems Compact X4. To carry out protein loading control, membrane was washed 4 times for 15 min in PBST and incubated for 1 h in 1:2000 GAPDH (Cell Signaling Technology) antibody in 5% milk. Following washing, membranes were incubated in 1:15,000 goat anti‐rabbit IgG HRP secondary antibody in 5% milk for 1 h and after incubation were washed for the final time. Membranes were incubated in ECL for 5 min, exposed to film and developed as before. Imaging of blots was performed using a BIORAD GS‐800 calibrated densitometer with QuantityOne 4.5.1 software (BIORAD) and band quantitation, normalised to a pooled standard and GAPDH, was carried out using ImagePro Plus 5.1.

### Statistical analyses

Statistical analysis was carried out using PASW Statistics 18.0. Baseline characteristics of each group were compared using an independent t‐test. Training effects on whole fibre measures were investigated using a repeated measures ANOVA with training and training mode as within and between group factors. In the case of significant main effects, pairwise comparisons were carried out using the Bonferroni post hoc test. For the analysis of GLUT4 in the PM layer and 1 *μ*m intracellular layers, a repeated measures ANOVA was used with training and layer as factors. In the case of a significant main effect of layer, pairwise comparisons were carried out using the Bonferroni post hoc test. In the case of a significant interaction between training and layer, paired *T* tests were carried out within layers to determine in which layers GLUT4 increased following training. Graphical data are expressed relative to pretraining values in type I fibres as mean ± SEM. For analysis of the PM layer and 1 *μ*m intracellular layers type I and type II fibres were not separated and graphical data are expressed relative to pretraining in the PM layer as mean ± SEM.

## Results

### Participant characteristics

Participant characteristics are displayed in Table 1. Sixteen sedentary age‐, BMI‐ and VO_2max_‐matched ET and SIT participants were insulin sensitive before the start of the training with no differences between groups. The full description of training adaptations including insulin sensitivity improvements have been previously reported (Cocks et al. 2013; Shepherd et al. 2013) with the same insulin sensitivity data as displayed in this paper. Insulin sensitivity, measured using the Matsuda insulin sensitivity index (Matsuda‐ISI), improved following 6 weeks of both ET and SIT (see Table 1).

**Table 1.** Subject characteristics before and after training

	ET, *N* = 8		SIT, *N* = 8	
	Pre	Post	Pre	Post
Age (year)	21 ± 1	–	22 ± 1	–
Height (m)	1.77 ± 0.03	1.77 ± 0.03	1.74 ± 0.02	1.74 ± 0.02
Body mass (kg)	70.8 ± 4.4	70.8 ± 4.5	75.1 ± 3.0	75.2 ± 3.1
BMI (kg·m^−2^)	22.6 ± 1.2	22.6 ± 1.2	24.8 ± 0.8	24.8 ± 0.9
VO_2max_ (mL·min^−1^·kg^−1^)	41.7 ± 4.1	48.2 ± 5.0*	41.9 ± 1.8	45.1 ± 2.3*
*W*_max_ (W)	218.5 ± 11.2	253.8 ± 16.0*	221.1 ± 11.4	241.5 ± 14.3*
Fasting plasma glucose (mmol/L^−1^)	5.6 ± 0.4	4.8 ± 0.6	4.9 ± 0.2	5.1 ± 0.2
2 hr OGTT plasma glucose (mmol/L^−1^)	7.0 ± 0.6	6.1 ± 0.3	6.3 ± 0.5	5.6 ± 0.4
Fasting plasma insulin (*μ*IU·mL^−1^)	12.3 ± 3.0	13.2 ± 3.2	13.1 ± 2.1	7.2 ± 0.5
HOMA‐IR	3.1 ± 0.7	2.7 ± 0.6	2.9 ± 0.5	1.6 ± 0.1
Matsuda‐ISI	3.7 ± 0.5	4.7 ± 0.7*	3.9 ± 0.3	5.8 ± 0.4*

Data displayed are mean ± SEM. Statistical significance was determined using a repeated measures ANOVA with post hoc Bonferroni analysis, pre‐ to post‐ training *P < 0.001.

### GLUT4 antibody validation

**Figure 2.**
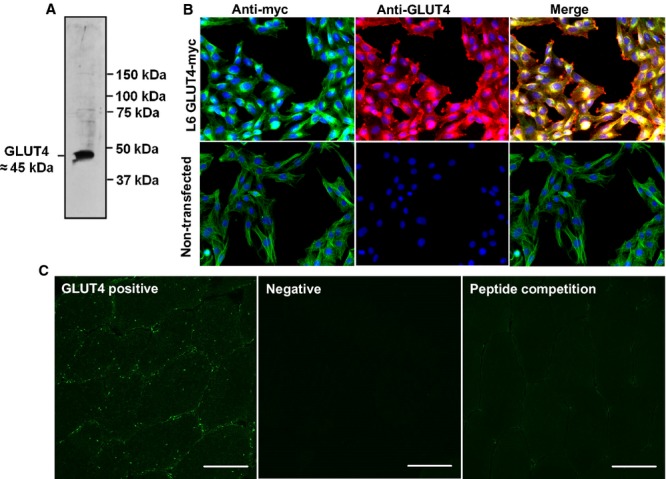
Validation of GLUT4 antibody. (A) Human skeletal muscle Western blot. (B) GLUT4‐myc transfected L6 cells (and non‐transfected) stained with GLUT4 antibody (red) and myc antibody (green). Myc staining is apparent in nontransfected L6 cells because they express endogenous myc (Denis et al. 1987). (C) Positive control. GLUT4 antibody staining for comparison to primary antibody omission negative and peptide competition staining. Scale bars 50 *μ*m.

The online UniProt database Basic Local Alignment Search Tool (BLAST; http://www.uniprot.org) indicated that the immunogen sequence corresponding to the C terminal 15 amino acid sequence of rat GLUT4 was not present in any protein other than GLUT4 in human skeletal muscle. Therefore an antibody raised against this immunogen sequence should only detect GLUT4 in human skeletal muscle sections. To confirm this experimentally Western blotting (Fig. 2A) was applied to human skeletal muscle and the GLUT4 antibody detected a single band at the expected molecular weight (approximately 45 kDa). Furthermore, this antibody has been used previously in publications for GLUT4 content estimates in whole muscle extracts in which the expected band of approximately 45 kDa was also detected (Cox et al. 2010; Stephenson et al. 2012). In L6 cells overexpressing GLUT4 with a myc tag (GLUT4‐myc), antibody staining against myc demonstrated extensive colocalisation with the staining generated by the GLUT4 antibody (Fig. 2B). Furthermore in nontransfected L6 cells, which exhibit very low expression of GLUT4, no positive GLUT4 staining was observed. The residual myc signal is attributable to endogenous myc in L6 cells (Denis et al. 1987). Incubation of the primary antibody with the immunogen peptide prior to application to the muscle section abolishes antibody staining, leaving only background fluorescence and confirming specificity of the antibody for the immunogen sequence (Fig. 2C, right panel). Omission of the GLUT4 primary antibody results in no staining, confirming secondary antibody specificity and absence of tissue autofluorescence (Fig. 2C, middle panel).

### Immunofluorescence visualisation of GLUT4 localisation in human skeletal muscle

**Figure 3.**
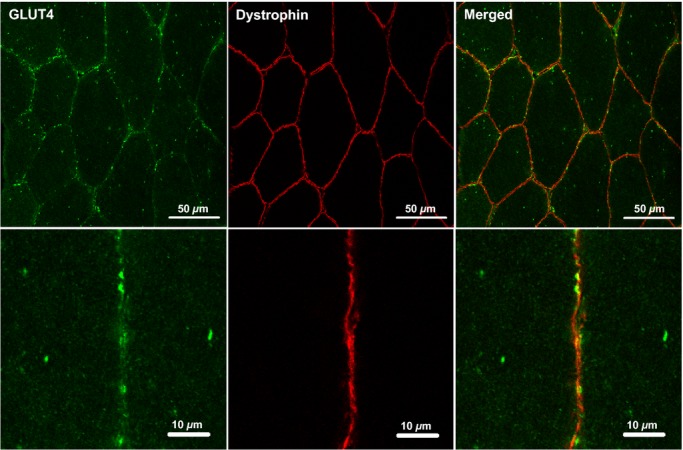
Representative confocal immunofluorescence microscopy images of GLUT4 (green) in human skeletal muscle stained in combination with anti‐dystrophin (red) to mark the PM. GLUT4 antibody is targeted with an AlexaFluor 488 goat anti‐rabbit IgG secondary antibody and dystrophin antibody is targeted with an AlexaFluor 594 goat anti‐mouse IgG_2b_ secondary antibody.

**Figure 4.**
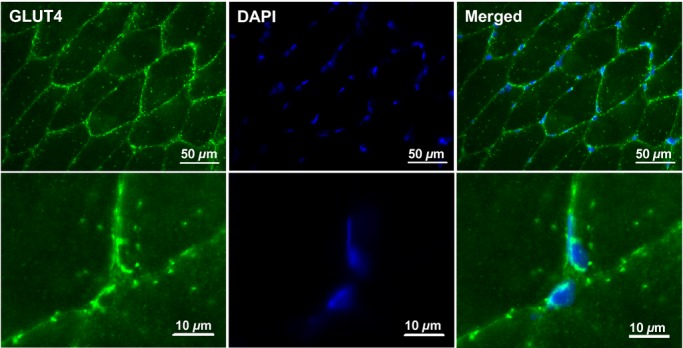
Representative widefield immunofluorescence microscopy images of GLUT4 (green) in human skeletal muscle stained in combination with DAPI (blue) to mark the cell nuclei.

**Figure 5.**
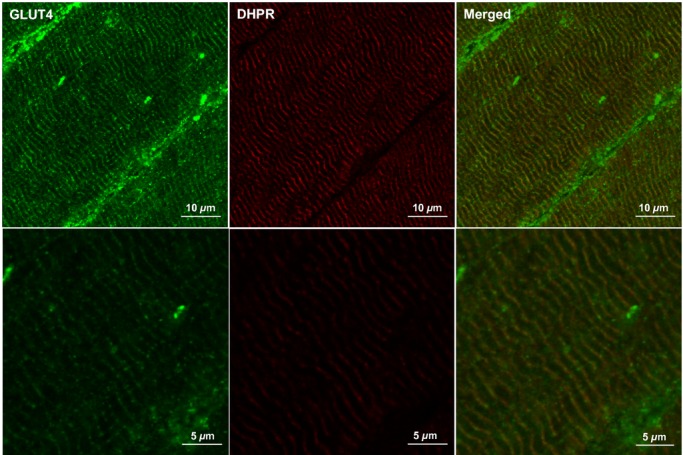
Representative confocal immunofluorescence microscopy images of GLUT4 (green) in human skeletal muscle stained in combination with anti‐DHPR (red) to mark the T tubule membranes. GLUT4 antibody is targeted with an AlexaFluor 488 goat anti‐rabbit IgG secondary antibody and DHPR antibody is targeted with an AlexaFluor 594 goat anti‐mouse IgG_2a_ secondary antibody.

**Figure 6.**
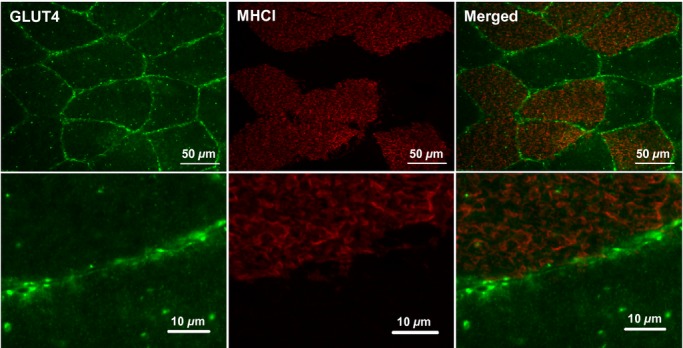
Representative widefield immunofluorescence microscopy images of GLUT4 (green) in human skeletal muscle stained in combination with anti‐MHCI (red) to mark the type I muscle fibres. GLUT4 antibody is targeted with an AlexaFluor 488 goat anti‐rabbit IgG secondary antibody and MHCI antibody is targeted with an AlexaFluor 594 goat anti‐mouse IgM secondary antibody.

**Figure 7.**
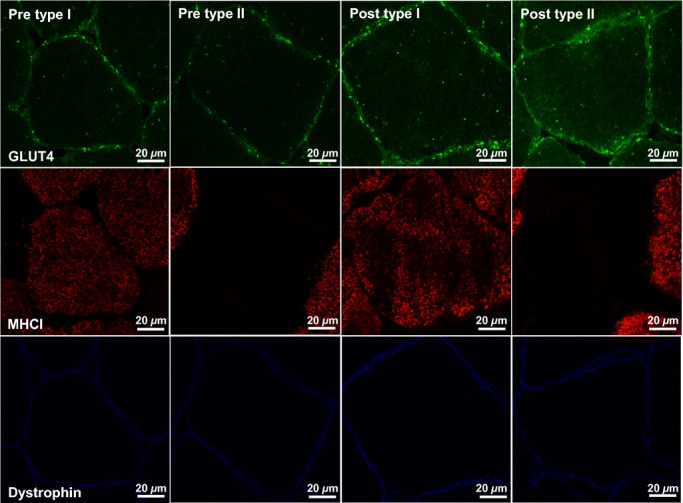
Representative confocal immunofluorescence images of type I and type II human skeletal muscle fibres in the basal state before and after ET. Images are also representative of SIT images. GLUT4 immunofluorescence staining (green) is combined with staining for MHC1 to mark type I fibres (red) and staining for dystrophin to mark the PM (blue). GLUT4 antibody is targeted with an AlexaFluor 488 goat anti‐rabbit IgG secondary antibody, MHCI antibody is targeted with an AlexaFluor 594 goat anti‐mouse IgM secondary antibody and dystrophin antibody is targeted with an AlexaFluor 633 goat anti‐mouse IgG_2b_ secondary antibody.

**Figure 8.**
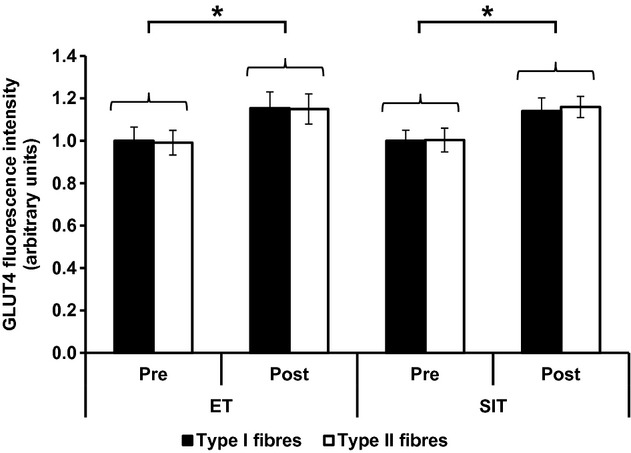
Total GLUT4 fluorescence intensity of type I and type II muscle fibres in the basal state before and after ET or SIT. Graph shows mean ± SEM (*n* = 8). Repeated measures ANOVA training effect *P = *0.018, training mode *P = *0.982, fibre type *P = *0.878. *P <**0.05.

Glucose transporter 4 staining in human skeletal muscle appears as large clusters of staining (>1 *μ*m) with smaller spots dispersed throughout the cell interior as can be seen in Figure 3. GLUT4 clusters are also observed close to or incorporated in the PM as indicated by the distinct colocalisation of GLUT4 with the PM marker dystrophin (Fig. 3). Another clear feature of human skeletal muscle GLUT4 localisation is a perinuclear distribution, which is shown in Figure 4 where GLUT4 staining surrounds the DAPI stain for cell nuclei. As well as staining in PM regions, Figure 5 shows that GLUT4 is also associated with the T‐tubule membranes in human skeletal muscle in the basal state. In longitudinally oriented muscle fibre sections stained for DHPR, a receptor found in high concentrations in the T‐tubule membranes and therefore used as a marker for the T‐tubule membranes, a striated pattern is seen in which the striations run perpendicular to the long axis of the fibre (Fig. 5, middle panel). GLUT4 staining demonstrates the same striation pattern (Fig. 5, left panel), which correspond to the DHPR striations when the two stains are overlaid (Fig. 5, right panel). In Figure 6 no difference in GLUT4 signal intensity is apparent between the type I muscle fibres, which positively stain for MHCI, and the type II muscle fibres. This visual observation is confirmed with quantitation in Figures 7 and 8.

### Immunofluorescence visualisation of GLUT4 content following exercise training

**Figure 9.**
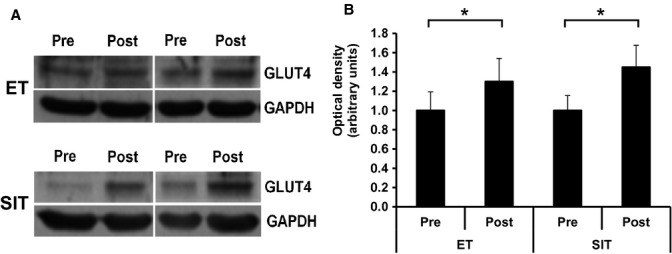
Representative Western blots of two ET and two SIT subjects showing skeletal muscle GLUT4 content before and after ET or SIT. GLUT4 content normalised to GAPDH and internal standard. Graph shows mean ± SEM (*n* = 8). Repeated measures ANOVA training effect *P = *0.05, training mode *P = *0.626, interaction *P = *0.43. *P <**0.05.

Representative confocal immunofluorescence images in Figure 7 show increased GLUT4 staining in both type I and type II muscle fibres following 6 weeks of training. GLUT4 fluorescence intensity increased with training (*P = *0.018) with no significant difference between training mode or muscle fibre type (Fig. 8). In type I fibres GLUT4 fluorescence intensity increased by 15 ± 6% following ET and 14 ± 5% following SIT. In type II fibres GLUT4 fluorescence intensity increased by 16 ± 6% following ET and 16 ± 5% following SIT. The increases in GLUT4 content using the immunofluorescence method were confirmed using Western blotting which detected a 30 ± 12% increase in GLUT4 content following ET and a 45 ± 27% increase following SIT (Fig. 9) (*P = *0.05).

### Training‐induced increases in intracellular GLUT4 stores

**Figure 10.**
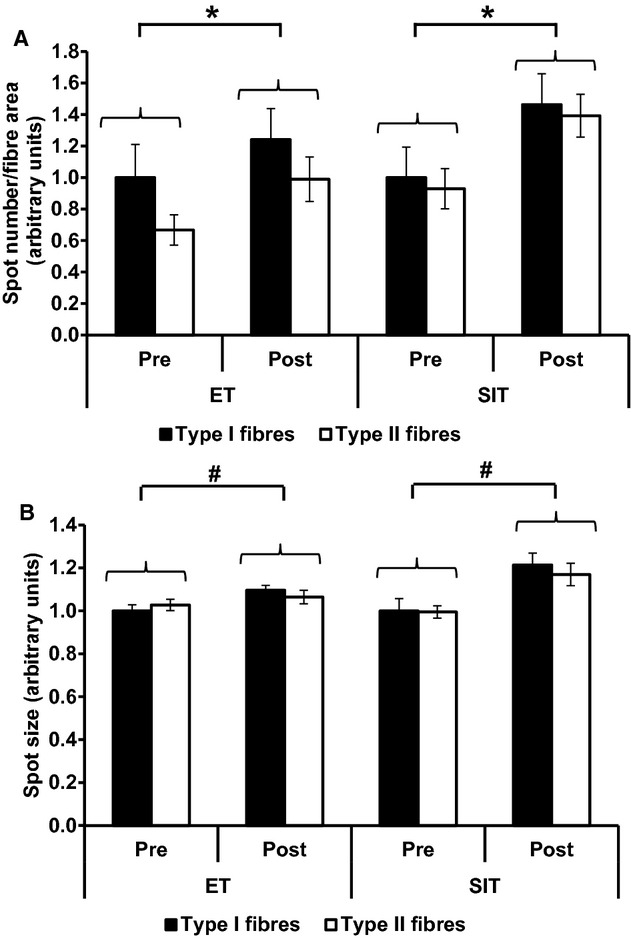
Quantitation of large spots of GLUT4 staining in type I and type II fibres in the basal state before and after ET or SIT. (A) Number of large spots per fibre area. Repeated measures ANOVA training effect *P = *0.002, training mode *P = *0.35, fibre type *P = *0.054. (B) Mean large spot size. Repeated measures ANOVA training effect *P = *0.011, training mode *P = *0.245, fibre type *P = *0.587. *P < 0.01, ^#^*P < *0.05. Graphs show mean ± SEM (*n* = 7).

**Figure 11.**
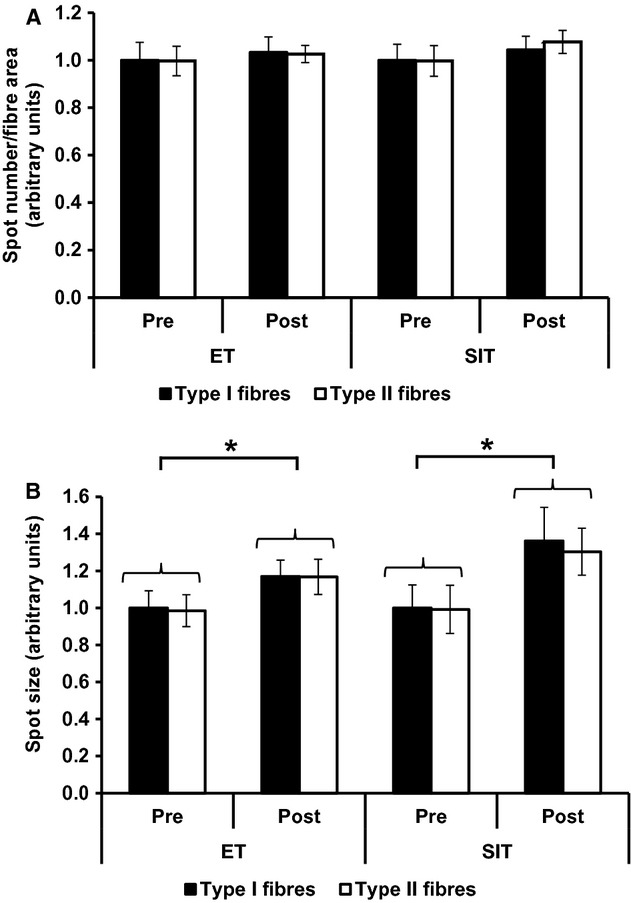
Quantitation of small spots of GLUT4 staining in type I and type II fibres in the basal state before and after ET or SIT. (A) Number of small spots per fibre area. Repeated measures ANOVA training *P = *0.230, training mode *P = *0.858, fibre type *P = *0.834. (B) Mean small spot size. Repeated measures ANOVA training effect *P = *0.028, training mode *P = *0.655, fibre type *P = *0.179. Graphs show mean ± SEM (*n* = 7). *P <**0.05.

GLUT4 immunofluorescence staining generates large bright clusters of GLUT4 staining as well as small stores which are dispersed throughout the cell interior (Fig. 3) and appear as a diffuse background stain in lower magnification images. Large and small spots can be identified by setting threshold limits on the spot sizes detected (>1 *μ*m or <1 *μ*m diameter, as in (Lauritzen et al. 2008)). Large intracellular spot number increased (*P = *0.002) following ET (24 ± 15% in type I fibres and 32 ± 6% in type II fibres) and SIT (46 ± 12% in type I fibres and 46 ± 13% in type II fibres) (Fig. 10A). The mean large spot size also increased (*P = *0.011) following ET (10 ± 3% in type I fibres and 4 ± 5% in type II fibres) and SIT (21 ± 88% in type I fibres and 17 ± 6% in type II fibres) (Fig. 10B). There was no change in the number of small intracellular spots following ET or SIT (Fig. 11A). However mean small spot size increased (*P = *0.028) following ET (17 ± 8% in type I fibres and 18 ± 8% in type II fibres) and SIT (36 ± 11% in type I fibres and 31 ± 11% in type II fibres) (Fig. 11B). There were no differences in the training response of the small and large GLUT4 spots between training modes (ET v SIT) or muscle fibre type (type I v type II muscle fibres).

### Quantitation of GLUT4 in the PM layer and 1 *μ*m intracellular layers

**Figure 12.**
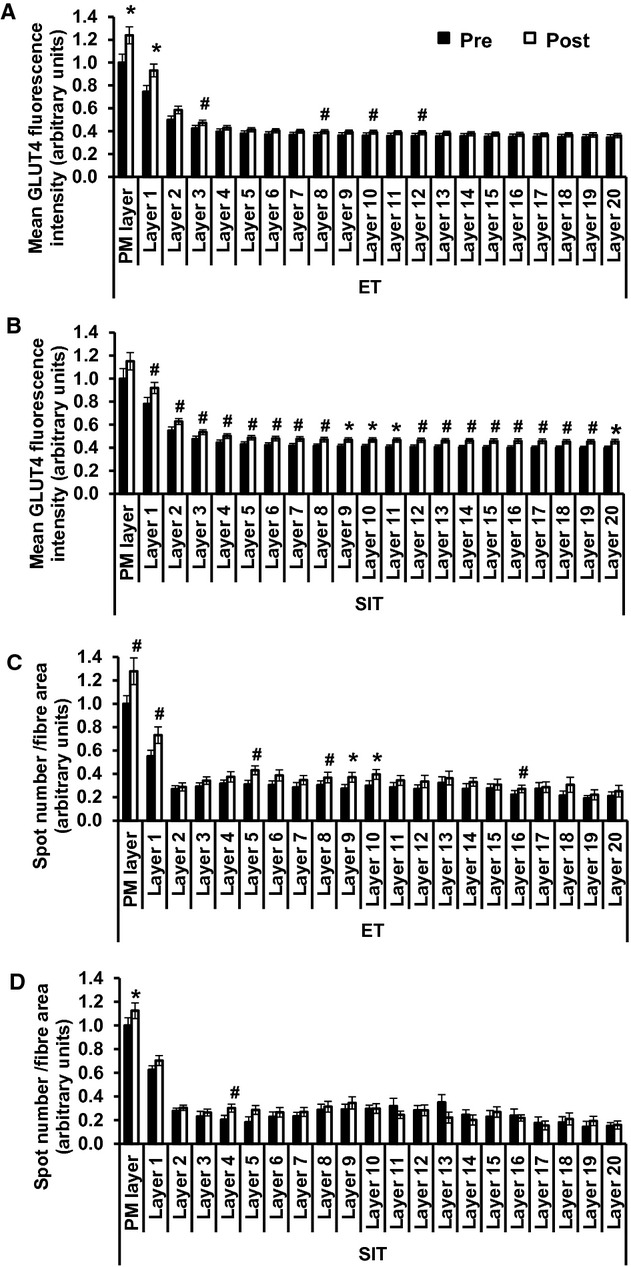
Quantitation of GLUT4 in the PM layer and 1 *μ*m layers progressing into the muscle fibre in the basal state before and after ET or SIT. (A) Mean GLUT4 fluorescence intensity ET. Repeated measures ANOVA training *P = *0.019, layer *P < *0.001, training*layer *P < *0.001. (B) Mean GLUT4 fluorescence intensity SIT. Repeated measures ANOVA training *P = *0.013, layer *P < *0.001, training*layer *P < *0.001. C) Large spot number/layer area ET. Repeated measures training *P = *0.011, layer *P < *0.001, training*layer *P = *0.002. (D) Large spot number/layer area SIT. Repeated measures ANOVA training *P = *0.334, layer *P < *0.001, training*layer *P = *0.018. Graphs show mean ± SEM (*n* = 8). Where there was a significant training*layer interaction, paired T tests were used to determine in which layers GLUT4 increased following training *P < 0.01, ^#^*P < *0.05.

Mean GLUT4 fluorescence intensity (repeated measures ANOVA ET training *P = *0.019, layer *P < *0.001, training*layer interaction *P < *0.001, Fig. 12A, SIT training *P = *0.013, layer *P < *0.001, training*layer interaction *P < *0.001, Fig. 12B) and large spot number (repeated measures ANOVA ET training *P = *0.011, layer *P < *0.001, training*layer interaction *P = *0.002, Fig. 12C, SIT training *P = *0.334, layer *P < *0.001, training*layer interaction *P = *0.018, Fig. 12D) were higher in the PM layer and first intracellular 1 *μ*m layer compared to intracellular layers in the basal state. Mean GLUT4 fluorescence intensity increased predominantly in the PM layer and first intracellular 1 *μ*m layer compared to subsequent intracellular layers following ET, while mean GLUT4 fluorescence intensity increased in all intracellular layers measured following SIT. The number of large spots normalised to layer area increased in the PM layer and first intracellular layer as well as five other intracellular layers following ET. Following SIT the number of large spots normalised to layer area increased only in the PM layer and fourth intracellular layer.

## Discussion

### GLUT4 localisation in human skeletal muscle

In this study an immunofluorescence microscopy method has been developed to investigate GLUT4 localisation and content in human skeletal muscle. GLUT4 immunofluorescence staining in human skeletal muscle in the basal state appears both as large bright clusters and smaller punctate spots, which are observed predominantly in the PM layer and perinuclear regions but also in intracellular layers. In addition GLUT4 staining demonstrates a striated pattern, which corresponds to the DHPR striations when the two stains are overlaid, therefore suggesting association of GLUT4 with the T‐tubule membranes in human skeletal muscle in the basal state. These results are in line with the distribution of the main GLUT4 compartments and transport vesicles described previously in rodent skeletal muscle (Ploug et al. 1998; Lauritzen et al. 2006, 2008). To investigate GLUT4 localisation with immunofluorescence microscopy, we rigorously validated a GLUT4 antibody to confirm antibody specificity and selectivity for GLUT4 protein using a combination of accepted techniques including Western blotting, GLUT4‐myc transfection of L6 cells, peptide competition and negative control experiments (Bordeaux et al. 2010).

### GLUT4 abundance following ET and SIT

In line with previous studies (Burgomaster et al. 2007; Little et al. 2010), 6 weeks of SIT and ET induced similar increases in the total protein content of GLUT4 in skeletal muscle. In addition a single bout of high‐ and low‐intensity exercise induced similar increases in total crude membrane GLUT4 protein content (Kraniou et al. 2006). The application of the novel immunofluorescence microscopy method in this study confirms the increase in GLUT4 protein expression and extends these findings by demonstrating that both modes of exercise increase GLUT4 expression in type I and type II muscle fibres. This increase in GLUT4 content likely contributes to the well described improvements in insulin‐mediated glucose uptake following both ET and SIT (Houmard et al. 1991; Hughes et al. 1993; Dela et al. 1996; Phillips et al. 1996; Cox et al. 1999; Daugaard et al. 2000; Kristiansen et al. 2000; Burgomaster et al. 2007; Babraj et al. 2009; Little et al. 2010; Richards et al. 2010; Hood et al. 2011).

### GLUT4 compartments in human skeletal muscle and their response to training

The increase in GLUT4 protein content following training in this study was due to an increase in the number and area of intracellular clusters of GLUT4. Large clusters (>1 *μ*m diameter) increased in number and size, while the smaller clusters (<1 *μ*m diameter) increased in size, with no increases in number. The large GLUT4 stores (>1 *μ*m) visualised here in human skeletal muscle are likely to be TGN‐associated GLUT4, as large GLUT4 structures have been identified as such in rodent skeletal muscle (Rodnick et al. 1992; Ploug et al. 1998). Studies in adipocytes report endosomal stores as approximately 150–200 nm and GSVs as 50–80 nm (Larance et al. 2008; Bogan and Kandror 2010; Stockli et al. 2011; Bogan 2012), while based on EM images endosomes appear to be in a similar range in rodent skeletal muscle (Ploug et al. 1998) and GSVs have been reported as 40 nm (Lizunov et al. 2012). Therefore, due to the resolution of confocal microscopy (~ 200 nm) the small stores detected here in human skeletal muscle could be endosomal membrane stores or GSVs, either individually or accumulations of smaller vesicles (Stockli et al. 2011). A greater TGN‐associated GLUT4 compartment as indicated in this study post‐training, may confer an insulin‐sensitivity advantage as this would increase the capacity for GSV budding at the TGN upon insulin stimulation (Larance et al. 2008; Bogan and Kandror 2010; Bogan 2012). The size of small GLUT4 stores also increased in response to training. If the small stores visualised here in human skeletal muscle are endosomal this will increase the capacity for GSV biogenesis at the endosome (Larance et al. 2008; Bogan and Kandror 2010; Bogan 2012). If the small stores are visible clouds of GSVs, this will increase the capacity for insulin‐mediated GLUT4 docking and fusion at the PM (Foley et al. 2011). GLUT4 immunofluorescence staining coupled to the targeted visualisation of established TGN and endosomal markers such as TGN38 and transferrin receptor (TfR), respectively, is required to generate harder evidence on the identity of the GLUT4 compartments observed here in human skeletal muscle.

### GLUT4 localisation in response to training

In this study image analysis confirmed mean GLUT4 fluorescence intensity and large GLUT4 spot number in the basal state were higher in the PM layer and the 1 *μ*m layer in from the PM compared to deeper layers. The high abundance of large GLUT4 spots in the PM layer and within 1 *μ*m of the PM in the basal state is consistent with data in rodent skeletal muscle showing that large clusters are present in sarcolemmal regions but remain stationary following insulin stimulation (Lauritzen et al. 2008). Lauritzen et al. (2008) suggested that these stationary vesicles release small GLUT4 vesicles (probably GSVs) which then translocate to and fuse with the PM. More recently, Lizunov et al. (2012) using TIRF imaging in live mouse muscle fibres generated evidence that stationary vesicles are pre‐tethered at the PM and that 80% of the total number of GLUT4‐PM fusion events actually emanated from these pre‐tethered vesicles. Therefore, considering the relatively stationary nature of the majority of GLUT4 compartments (Lauritzen et al. 2008; Lizunov et al. 2012), the high density of GLUT4 present in the PM layer and 1 *μ*m layers immediately adjacent to the PM means that a large proportion of GLUT4 is advantageously located in a position ready for fusion with the PM upon insulin stimulation. Furthermore, mean GLUT4 fluorescence intensity and large spot number increased consistently in the PM layer and first 1 *μ*m intracellular compared to deeper layers following training. As already explained, higher GLUT4 in PM regions may confer an insulin‐sensitivity advantage and could provide the link between greater GLUT4 protein content and greater insulin‐mediated GLUT4 translocation and improved insulin sensitivity post‐training.

This image analysis approach is a novel tool to investigate changes in the main skeletal muscle GLUT4 stores in response to acute exercise and training, focussing on the PM layer and first intracellular layers from which fusion events emanate. This level of information regarding GLUT4 stores and localisation following training is the maximum achieved to date in human skeletal muscle using standard confocal microscopy techniques.

### Study limitations

A single fibre was captured per image in order to achieve sufficient resolution to generate the valuable information on the GLUT4 content of the PM and 1 *μ*m layers below the PM. Therefore the number of fibres analysed was compromised in favour of resolution of the images, meaning a restricted number of fibres were analysed in this study. While we acknowledge the restricted fibre number is a limitation of the study, it is clear that we do observe significant increases in specific locations with 5 type I and 5 type II fibres per section.

## Conclusions

This study has employed a novel immunofluorescence method with a validated antibody to visualise training (ET and SIT) induced changes in the subcellular distribution and content of GLUT4. The results show that comparable increases in total skeletal muscle GLUT4 content occur following the two training modes through increases in the number and size of large GLUT4 clusters (>1 *μ*m; including TGN stores) and increases in size of the smaller GLUT4 clusters (<1 *μ*m; including endosomal membranes and GSVs). Furthermore mean GLUT4 fluorescence intensity and large spot number was higher in the PM layer and first intracellular layer compared to deeper layers and increased to a greater extent within the PM layer and 1 *μ*m layer immediately adjacent to the PM compared to deeper regions. The increases in whole body insulin sensitivity observed following the two training modes (Table 1) and the training induced increases in insulin‐stimulated skeletal muscle glucose uptake in humans (Dela et al. 1992, 1995, 1996; Kristiansen et al. 2000; Frosig et al. 2007) are in part driven by this increase in the content of the GLUT4 storage compartments. This novel analytical approach has the potential to substantially improve our understanding of the role that impairments in GLUT4 fusion with the PM play in the impaired glucose homeostasis that occur in chronic age and lifestyle‐related metabolic diseases and will be a useful endpoint for future training studies in sedentary populations with and without insulin resistance.

## Acknowledgements

The antibody against myosin (human slow twitch fibres, A4.840) used in the study was developed by Dr. Blau, and obtained from the Developmental Studies Hybridoma Bank developed under the auspices of the NICHD and maintained by the University of Iowa, Department of Biological Sciences, Iowa City, IA 52242. We are very grateful to Dr Samantha Peel for assisting with GLUT4‐myc L6 cells experiments carried out onsite at AstraZeneca. We thank A.M. Ranasinghe and T.A. Barker of the School of Clinical and Experimental Medicine, University of Birmingham, for their support in the collection of muscle biopsy samples.

## Conflict of Interest

This study was funded by AstraZeneca, UK. In addition, H.B. was funded by a studentship from AstraZeneca, UK.

## References

[b1] Babraj J. A. Vollaard N. B. Keast C. Guppy F. M. Cottrell G. Timmons J. A. 2009 Extremely short duration high intensity interval training substantially improves insulin action in young healthy males. BMC Endo. Disord.; 9:3 10.1186/1472-6823-9-3 PMC2640399 19175906

[b2] Bell G. I. Burant C. F. Takeda J. Gould G. W. 1993 Structure and function of mammalian facilitative sugar transporters. J. Biol. Chem.; 268:19161-19164. 8366068

[b3] Bergstrom J. 1975 Percutaneous needle biopsy of skeletal muscle in physiological and clinical research. Scand. J. Clin. Lab. Invest.; 35:609-616. 1108172

[b4] Bogan J. S. 2012 Regulation of glucose transporter translocation in health and diabetes. Annu. Rev. Biochem.; 81:507-532. 22482906 10.1146/annurev-biochem-060109-094246

[b5] Bogan J. S. Kandror K. V. 2010 Biogenesis and regulation of insulin‐responsive vesicles containing GLUT4. Curr. Opin. Cell Biol.; 22:506-512. 20417083 10.1016/j.ceb.2010.03.012 PMC2910140

[b6] Bordeaux J. Welsh A. Agarwal S. Killiam E. Baquero M. Hanna J. 2010 Antibody validation. Biotechniques; 48:197-209. 20359301 10.2144/000113382 PMC3891910

[b7] Burgomaster K. A. Cermak N. M. Phillips S. M. Benton C. R. Bonen A. Gibala M. J. 2007 Divergent response of metabolite transport proteins in human skeletal muscle after sprint interval training and detraining. Am. J. Physiol. Regul. Integr. Comp. Physiol.; 292:R1970-R1976. 17303684 10.1152/ajpregu.00503.2006

[b8] Cocks M. Shaw C. S. Shepherd S. O. Fisher J. P. Ranasinghe A. M. Barker T. A. 2013 Sprint interval and endurance training are equally effective in increasing muscle microvascular density and eNOS content in sedentary males. J. Physiol.; 591:641-656. 22946099 10.1113/jphysiol.2012.239566 PMC3577551

[b9] Cox J. H. Cortright R. N. Dohm G. L. Houmard J. A. 1999 Effect of aging on response to exercise training in humans: skeletal muscle GLUT‐4 and insulin sensitivity. J. Appl. Physiol.; 86:2019-2025. 10368369 10.1152/jappl.1999.86.6.2019

[b10] Cox G. R. Clark S. A. Cox A. J. Halson S. L. Hargreaves M. Hawley J. A. 2010 Daily training with high carbohydrate availability increases exogenous carbohydrate oxidation during endurance cycling. J. Appl. Physiol.; 109:126-134. 20466803 10.1152/japplphysiol.00950.2009

[b11] Daugaard J. R. Nielsen J. N. Kristiansen S. Andersen J. L. Hargreaves M. Richter E. A. 2000 Fiber type‐specific expression of GLUT4 in human skeletal muscle: influence of exercise training. Diabetes; 49:1092-1095. 10909963 10.2337/diabetes.49.7.1092

[b12] Dela F. Mikines K. J. von Linstow M. Secher N. H. Galbo H. 1992 Effect of training on insulin‐mediated glucose uptake in human muscle. Am. J. Physiol.; 263:E1134-E1143. 1476187 10.1152/ajpendo.2006.263.6.E1134

[b13] Dela F. Larsen J. J. Mikines K. J. Ploug T. Petersen L. N. Galbo H. 1995 Insulin‐stimulated muscle glucose clearance in patients with NIDDM. Effects of one‐legged physical training. Diabetes; 44:1010-1020. 7657022 10.2337/diab.44.9.1010

[b14] Dela F. Mikines K. J. Larsen J. J. Galbo H. 1996 Training‐induced enhancement of insulin action in human skeletal muscle: the influence of aging. J. Gerontol. Series A, Biol. Sci. Med. Sci.; 51:B247-B252. 10.1093/gerona/51a.4.b247 8680988

[b15] Denis N. Blanc S. Leibovitch M. P. Nicolaiew N. Dautry F. Raymondjean M. 1987 c‐myc oncogene expression inhibits the initiation of myogenic differentiation. Exp. Cell Res.; 172:212-217. 2443373 10.1016/0014-4827(87)90107-8

[b16] Douen A. G. Ramlal T. Cartee G. D. Klip A. 1990 Exercise modulates the insulin‐induced translocation of glucose transporters in rat skeletal muscle. FEBS Lett.; 261:256-260. 2178971 10.1016/0014-5793(90)80566-2

[b17] Fazakerley D. J. Lawrence S. P. Lizunov V. A. Cushman S. W. Holman G. D. 2009 A common trafficking route for GLUT4 in cardiomyocytes in response to insulin, contraction and energy‐status signalling. J. Cell Sci.; 122:727-734. 19208760 10.1242/jcs.041178 PMC2720923

[b18] Foley K. Boguslavsky S. Klip A. 2011 Endocytosis, recycling, and regulated exocytosis of glucose transporter 4. Biochemistry; 50:3048-3061. 21405107 10.1021/bi2000356

[b19] Frosig C. Rose A. J. Treebak J. T. Kiens B. Richter E. A. Wojtaszewski J. F. 2007 Effects of endurance exercise training on insulin signaling in human skeletal muscle: interactions at the level of phosphatidylinositol 3‐kinase, Akt, and AS160. Diabetes; 56:2093-2102. 17513702 10.2337/db06-1698

[b20] Goodyear L. J. Hirshman M. F. Napoli R. Calles J. Markuns J. F. Ljungqvist O. 1996 Glucose ingestion causes GLUT4 translocation in human skeletal muscle. Diabetes; 45:1051-1056. 8690151 10.2337/diab.45.8.1051

[b21] Hansen P. A. Gulve E. A. Marshall B. A. Gao J. Pessin J. E. Holloszy J. O. 1995 Skeletal muscle glucose transport and metabolism are enhanced in transgenic mice overexpressing the Glut4 glucose transporter. J. Biol. Chem.; 270:1679-1684. 7829503 10.1074/jbc.270.5.1679

[b22] Hood M. S. Little J. P. Tarnopolsky M. A. Myslik F. Gibala M. J. 2011 Low‐volume interval training improves muscle oxidative capacity in sedentary adults. Med. Sci. Sports Exerc.; 43:1849-1856. 21448086 10.1249/MSS.0b013e3182199834

[b23] Houmard J. A. Egan P. C. Neufer P. D. Friedman J. E. Wheeler W. S. Israel R. G. 1991 Elevated skeletal muscle glucose transporter levels in exercise‐trained middle‐aged men. Am. J. Physiol.; 261:E437-E443. 1928336 10.1152/ajpendo.1991.261.4.E437

[b24] Hughes V. A. Fiatarone M. A. Fielding R. A. Kahn B. B. Ferrara C. M. Shepherd P. 1993 Exercise increases muscle GLUT‐4 levels and insulin action in subjects with impaired glucose tolerance. Am. J. Physiol.; 264:E855-E862. 8333511 10.1152/ajpendo.1993.264.6.E855

[b25] Kanai F. Nishioka Y. Hayashi H. Kamohara S. Todaka M. Ebina Y. 1993 Direct demonstration of insulin‐induced GLUT4 translocation to the surface of intact cells by insertion of a c‐myc epitope into an exofacial GLUT4 domain. J. Biol. Chem.; 268:14523-14526. 7686158

[b26] Kass M. Witkin A. Terzopoulos D. 1988 Snakes: Active contour models. Int. J. Comput. Vis.; 1:321-331.

[b27] Katz L. D. Glickman M. G. Rapoport S. Ferrannini E. DeFronzo R. A. 1983 Splanchnic and peripheral disposal of oral glucose in man. Diabetes; 32:675-679. 6862113 10.2337/diab.32.7.675

[b28] Kennedy J. W. Hirshman M. F. Gervino E. V. Ocel J. V. Forse R. A. Hoenig S. J. 1999 Acute exercise induces GLUT4 translocation in skeletal muscle of normal human subjects and subjects with type 2 diabetes. Diabetes; 48:1192-1197. 10331428 10.2337/diabetes.48.5.1192

[b29] Klip A. Ramlal T. Young D. A. Holloszy J. O. 1987 Insulin‐induced translocation of glucose transporters in rat hindlimb muscles. FEBS Lett.; 224:224-230. 2960560 10.1016/0014-5793(87)80452-0

[b30] Kraniou G. N. Cameron‐Smith D. Hargreaves M. 2006 Acute exercise and GLUT4 expression in human skeletal muscle: influence of exercise intensity. J. Appl. Physiol.; 101:934-937. 16763099 10.1152/japplphysiol.01489.2005

[b31] Kristiansen S. Gade J. Wojtaszewski J. F. Kiens B. Richter E. A. 2000 Glucose uptake is increased in trained vs. untrained muscle during heavy exercise. J. Appl. Physiol.; 89:1151-1158. 10956363 10.1152/jappl.2000.89.3.1151

[b32] Larance M. Ramm G. James D. E. 2008 The GLUT4 code. Mol. Endocrinol.; 22:226-233. 17717074 10.1210/me.2007-0282 PMC5419637

[b33] Lauritzen H. P. Ploug T. Prats C. Tavare J. M. Galbo H. 2006 Imaging of insulin signaling in skeletal muscle of living mice shows major role of T‐tubules. Diabetes; 55:1300-1306. 16644686 10.2337/db05-1216

[b34] Lauritzen H. P. Galbo H. Brandauer J. Goodyear L. J. Ploug T. 2008 Large GLUT4 vesicles are stationary while locally and reversibly depleted during transient insulin stimulation of skeletal muscle of living mice: imaging analysis of GLUT4‐enhanced green fluorescent protein vesicle dynamics. Diabetes; 57:315-324. 17977960 10.2337/db06-1578

[b35] Leturque A. Loizeau M. Vaulont S. Salminen M. Girard J. 1996 Improvement of insulin action in diabetic transgenic mice selectively overexpressing GLUT4 in skeletal muscle. Diabetes; 45:23-27. 8522055 10.2337/diab.45.1.23

[b36] Little J. P. Safdar A. Wilkin G. P. Tarnopolsky M. A. Gibala M. J. 2010 A practical model of low‐volume high‐intensity interval training induces mitochondrial biogenesis in human skeletal muscle: potential mechanisms. J. Physiol.; 588:1011-1022. 20100740 10.1113/jphysiol.2009.181743 PMC2849965

[b37] Lizunov V. A. Stenkula K. G. Lisinski I. Gavrilova O. Yver D. R. Chadt A. 2012 Insulin stimulates fusion, but not tethering, of GLUT4 vesicles in skeletal muscle of HA‐GLUT4‐GFP transgenic mice. Am. J. Physiol. Endocrinol. Metab.; 302:E950-E960. 22297303 10.1152/ajpendo.00466.2011 PMC3330721

[b38] Marette A. Richardson J. M. Ramlal T. Balon T. W. Vranic M. Pessin J. E. 1992 Abundance, localization, and insulin‐induced translocation of glucose transporters in red and white muscle. Am. J. Physiol.; 263:C443-C452. 1514590 10.1152/ajpcell.1992.263.2.C443

[b39] Mueckler M. 1994 Facilitative glucose transporters. Eur. J. Biochem.; 219:713-725. 8112322 10.1111/j.1432-1033.1994.tb18550.x

[b40] Phillips S. M. Han X. X. Green H. J. Bonen A. 1996 Increments in skeletal muscle GLUT‐1 and GLUT‐4 after endurance training in humans. Am. J. Physiol.; 270:E456-E462. 8638693 10.1152/ajpendo.1996.270.3.E456

[b41] Ploug T. Van Deurs B. Ai H. Cushman S. W. Ralston E. 1998 Analysis of GLUT4 distribution in whole skeletal muscle fibers: identification of distinct storage compartments that are recruited by insulin and muscle contractions. J. Cell Biol.; 142:1429-1446. 9744875 10.1083/jcb.142.6.1429 PMC2141761

[b42] Ren J. M. Marshall B. A. Mueckler M. M. McCaleb M. Amatruda J. M. Shulman G. I. 1995 Overexpression of Glut4 protein in muscle increases basal and insulin‐stimulated whole body glucose disposal in conscious mice. J. Clin. Investig.; 95:429-432. 7814644 10.1172/JCI117673 PMC295454

[b43] Richards J. C. Johnson T. K. Kuzma J. N. Lonac M. C. Schweder M. M. Voyles W. F. 2010 Short‐term sprint interval training increases insulin sensitivity in healthy adults but does not affect the thermogenic response to beta‐adrenergic stimulation. J. Physiol.; 588:2961-2972. 20547683 10.1113/jphysiol.2010.189886 PMC2956910

[b44] Rodnick K. J. Slot J. W. Studelska D. R. Hanpeter D. E. Robinson L. J. Geuze H. J. 1992 Immunocytochemical and biochemical studies of GLUT4 in rat skeletal muscle. J. Biol. Chem.; 267:6278-6285. 1556135

[b45] Schertzer J. D. Antonescu C. N. Bilan P. J. Jain S. Huang X. Liu Z. 2009 A transgenic mouse model to study glucose transporter 4myc regulation in skeletal muscle. Endocrinology; 150:1935-1940. 19074577 10.1210/en.2008-1372

[b46] Shepherd S. O. Cocks M. Tipton K. D. Ranasinghe A. M. Barker T. A. Burniston J. G. 2013 Sprint interval and traditional endurance training increase net intramuscular triglyceride breakdown and expression of perilipin 2 and 5. J. Physiol.; 591:657-675. 23129790 10.1113/jphysiol.2012.240952 PMC3577544

[b47] Stephenson E. J. Stepto N. K. Koch L. G. Britton S. L. Hawley J. A. 2012 Divergent skeletal muscle respiratory capacities in rats artificially selected for high and low running ability: a role for Nor1? J. Appl. Physiol.; 113:1403-1412. 22936731 10.1152/japplphysiol.00788.2012 PMC3524666

[b48] Stockli J. Fazakerley D. J. James D. E. 2011 GLUT4 exocytosis. J. Cell Sci.; 124:4147-4159. 22247191 10.1242/jcs.097063 PMC3258103

[b49] Tsao T. S. Burcelin R. Katz E. B. Huang L. Charron M. J. 1996 Enhanced insulin action due to targeted GLUT4 overexpression exclusively in muscle. Diabetes; 45:28-36. 8522056 10.2337/diab.45.1.28

[b50] Wang Q. Khayat Z. Kishi K. Ebina Y. Klip A. 1998 GLUT4 translocation by insulin in intact muscle cells: detection by a fast and quantitative assay. FEBS Lett.; 427:193-197. 9607310 10.1016/s0014-5793(98)00423-2

[b51] Wasserman D. H. Kang L. Ayala J. E. Fueger P. T. Lee‐Young R. S. 2011 The physiological regulation of glucose flux into muscle in vivo. J. Exp. Biol.; 214:254-262. 21177945 10.1242/jeb.048041 PMC3008632

